# Chikungunya Outbreak in Bueng Kan Province, Thailand, 2013

**DOI:** 10.3201/eid2008.140481

**Published:** 2014-08

**Authors:** Nasamon Wanlapakorn, Thanunrat Thongmee, Piyada Linsuwanon, Paiboon Chattakul, Sompong Vongpunsawad, Sunchai Payungporn, Yong Poovorawan

**Affiliations:** Chulalongkorn University, Bangkok, Thailand (N. Wanlapakorn, T. Thongmee, P. Linsuwanon, S. Vongpunsawad, S. Payungporn, Y. Poovorawan);; Bueng Kan Provincial Hospital, Bueng Kan, Thailand (P. Chattakul)

**Keywords:** chikungunya, viruses, Thailand, Laos, Cambodia, phylogenetic analysis, *Aedes albopictus*, *Aedes aegypti*, mosquito, vectorborne, CHIKV

**To the Editor:** Chikungunya fever is a dengue-like syndrome characterized by acute fever, arthralgia, and maculopapular rash. The causative agent is chikungunya virus (CHIKV), which is transmitted by *Aedes aegypti* and *Aedes albopictus* mosquitoes (*1*). Based on the genome and the viral envelope E1 sequences, CHIKV is classified into 3 genetic lineages: Asian, West African, and East/Central/South African (ECSA) genotypes (*2*).

In Thailand, the first report of CHIKV infection occurred in Bangkok in 1958 (*3*); later, sporadic cases of chikungunya fever occurred in many provinces during 1976–1995 (*4*). All of the CHIKV strains found in Thailand at that time were of the Asian genotype. The virus has since reemerged during 2008–2009 and caused large outbreaks in southern Thailand, affecting >50,000 persons (*5*). These outbreaks were attributed to the ECSA genotype. We report an outbreak of CHIKV infection in the northeastern province of Bueng Kan in 2013.

Bueng Kan Province is located on the Mekong River on the foothills of the mountainous region of Laos to the north. An outbreak of suspected dengue cases was reported during the rainy season during April–September 2013(*6*). Beginning in September, however, hospital physicians noticed that patients were reporting fever with moderate to severe joint pain resulting in limitation of movement that lasted for weeks. Serum samples were collected from 109 persons (hospitalized and outpatient) in October. Clinical data showed that 38 (34.9%) had moderate to severe joint pain; median duration of illness was 4 days (range 1–7). Median timing of sample collection from the onset of illness was 8 days (range 1–21).

Samples were sent to Chulalongkorn University Hospital in Bangkok to screen for mosquitoborne viruses. The study protocol was approved by the Institutional Review Board of Chulalongkorn University and consents were waived because all samples were stored as anonymous. Viral genomic RNA was assayed by using seminested reverse transcription PCR (RT-PCR) for CHIKV nucleic acid (*7*). Serum samples were tested for IgM antibodies against CHIKV by using SD BIOLINE Chikungunya IgM Test (Standard Diagnostics Inc., Kyonggi-do, South Korea) (*8*). In our study, the criteria for diagnosis of CHIKV infection included the detection of CHIKV nucleic acid by RT-PCR or IgM antibodies against CHIKV.

Of the 109 samples tested, 51 (46.8%) had evidence of CHIKV infection, as 25 (22.9%) were positive for CHIKV RNA by RT-PCR, and 32 (29.3%) were positive for IgM antibodies. Both CHIKV nucleic acid and IgM were found in 6 samples. To further characterize the phylogenetic relationships between the CHIKV strains in this outbreak with strains previously found in Thailand and neighboring regions, we performed full-length viral genomic sequencing from srains from 4 samples by primer walking (*4*). We subjected sequences to BLAST analysis (BLAST, http://blast.ncbi.nlm.nih.gov), aligned using the BioEdit program, v7.1.9 (http://www.mbio.ncsu.edu/bioedit/bioedit.html) and performed sequence assembly using the DNASTAR v6.0 (DNA Star, Madison, WI, USA). We performed maximum likelihood phylogenetic analysis of a set of 11,710 nt, including the sequences identified in this study (THA/Bueng Kan/BK46/2013, THA/Bueng Kan/BK57/2013, THA/Bueng Kan/BK63/2013, THA/Bueng Kan/BK68/2013; accession nos. KJ579184–7) by using the MEGA program v6.0 (http://www.megasoftware.net). Taking into consideration possible co-circulation of the Asian and ECSA genotypes in Thailand, we also examined intergenotypic recombination using the Recombination Detection Program, v4.22 (http://en.bio-soft.net/tree/RDP.html).

Phylogenetic analysis, comparing the 4 strains of CHIKV identified from Bueng Kan to 52 additional whole genomic sequences from GenBank, revealed that the 4 strains are closely related and share >99.8% pairwise nucleotide identity (Figure). The Bueng Kan isolates grouped to the ECSA genotype within the recent Indian Ocean clade (*9*). This relationship was confirmed by analysis of the nonstructural, structural, and E1 encoding regions (data not shown). The THA/Bueng Kan strains all possessed an alanine to valine change at residue 226 (A226V) in the E1 gene, which was noted as one of the crucial substitutions for increased transmissibility by *Aedes albopictus* reported for the Réunion Island isolates (*9*). This substitution was shared among strains isolated in Thailand in 2008, which were responsible for the previous CHIKV outbreaks in southern Thailand (*7*), and those in the recent outbreak in Cambodia (*10*). This suggests the presence and continued circulation of the E1-A226V virus in Thailand since 2008. However, another mutation specific to the Indian Ocean isolates, D284E in the E1 gene, was not found in THA/Bueng Kan strains. No evidence of recombination has been found in the THA/Bueng Kan strains and other strains identified in Thailand thus far.

**Figure:**
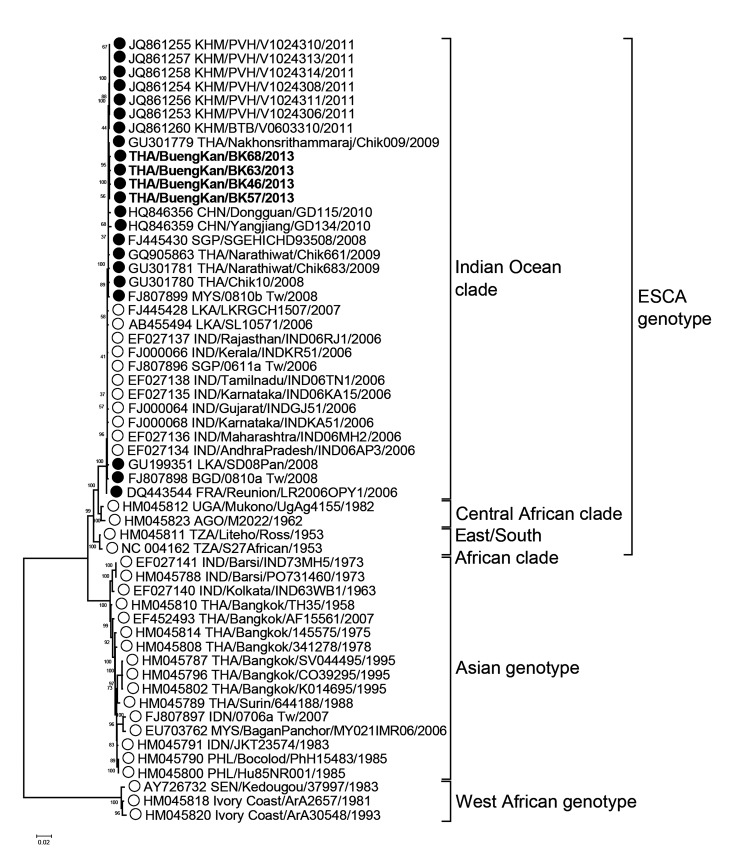
Phylogenetic analysis of whole genome nucleotide sequences of chikungunya virus (CHIKV) isolated during the 2013 outbreak in Bueng Kan Province, Thailand. The trees were generated by maximum-likelihood method, and the numbers along the branches indicate bootstrap values. Scale bar denotes nucleotide substitutions per site. Branch support and nodal confidence was assessed by using a general time reversible +I^−^4 nt substitution model with 1,000 bootstrap resampling. All sequences are labeled with GenBank accession number, country (3 letter code) and city of origin, strain name, and year of sampling. Bold text indicates CHIKV isolates identified in this study. Black and white circles on the tree indicate E1-A226V mutant and nonmutant strains, respectively.

Viral infections by mosquitoes continue to challenge public health in Thailand. In evidence of this, we demonstrated that CHIKV has become established in northeastern Thailand. Surveillance and clinical recognition will not prevent future outbreaks, but rather will assist in organizing an early response to outbreaks and thus minimize unnecessary illness and death.
